# Cervical vagus nerve morphometry and vascularity in the context of nerve stimulation - A cadaveric study

**DOI:** 10.1038/s41598-018-26135-8

**Published:** 2018-05-22

**Authors:** Niels Hammer, Sabine Löffler, Yusuf Ozgur Cakmak, Benjamin Ondruschka, Uwe Planitzer, Michael Schultz, Dirk Winkler, David Weise

**Affiliations:** 1 Department of Anatomy, University of Otago, Dunedin, New Zealand; 2 Department of Anatomy, University of Leipzig, Leipzig, Germany; 3 Institute of Legal Medicine, University of Leipzig, Leipzig, Germany; 4 Department of Neurosurgery, University of Leipzig, Leipzig, Germany; 5 Department of Medicine, Dunedin School of Medicine, University of Otago, Dunedin, New Zealand; 6 Gastroenterology Unit, Southern District Health Board, Dunedin Hospital, Dunedin, New Zealand; 7 Department of Neurology, University of Leipzig, Leipzig, Germany; 8 Department of Orthopaedic, Trauma and Plastic Surgery, University of Leipzig, Leipzig, Germany

## Abstract

Vagus nerve stimulation (VNS) has become a well-established therapy for epilepsy and depression, and is emerging to treat inflammatory disease, with the cervical vagus nerve (CVN) as major stimulation site. CVN morphometries are missing for VNS, considering its variability. Morphometric data were obtained from CVNs in 27 cadavers, including branching patterns and histology. Cross-sectional area, greater and lesser diameters averaged 7.2 ± 3.1 mm^2^, 5.1 ± 1.5 and 4.1 ± 1.3 mm, and were ≤11.0 mm^2^, ≤7.0 and ≤5.8 mm in 90% of the specimens, respectively. Midline distance (position lateral to the laryngeal eminence) and skin distance (anterior-posterior from skin) averaged 34.5 ± 6.2 and 36.2 ± 9.4 mm, ≤49.0 and ≤41.0 mm in 90%, respectively. Nerve dimensions and surface topography correlated closely, but without gender-, side- or branching-dependent differences. The nerve fascicle number averaged 5.2 ± 3.5. Vagal arteries were observed in 49% of the cases. Negative correlations were found for age and cross-sectional area, as well as subperineural vessel count. Detailed anatomical data on the CVN and its vascularity are given, forming the morphometric basis for VNS refinement, filling an evident gap in light of the CVN being a structure with variable positions and branching. A 35 × 35-mm rule may apply for the CVN position, irrespective of branching or positional variation.

## Introduction

Vagus nerve stimulation (VNS) has become a well-established therapy for drug-resistant epilepsy^1–5^ and refractory depression^2,6^, and is an emerging treatment option for inflammatory bowel disease (IBD)^7–12^. Further indications for VNS are currently studied, including potential effects on the immune system and inflammation^11,13^. Even if VNS is performed frequently, therapeutic effects are variable and side effects quite common^2,14–16^.

The cervical vagus nerve (CVN) is composed of both parasympathetic and sympathetic fibers supplying the heart^17,18^, lungs and abdominal viscera^19–21^. A major portion of the CVN is composed of afferent fibers^22^, likely related to VNS effects. The underlying mechanisms for the therapeutic effects are known to be closely related to the nucleus tractus solitarius, dorsal motor nucleus of vagus and their peripheral and supraspinal connections to various other cortical structures^23^.

**Figure 1:**
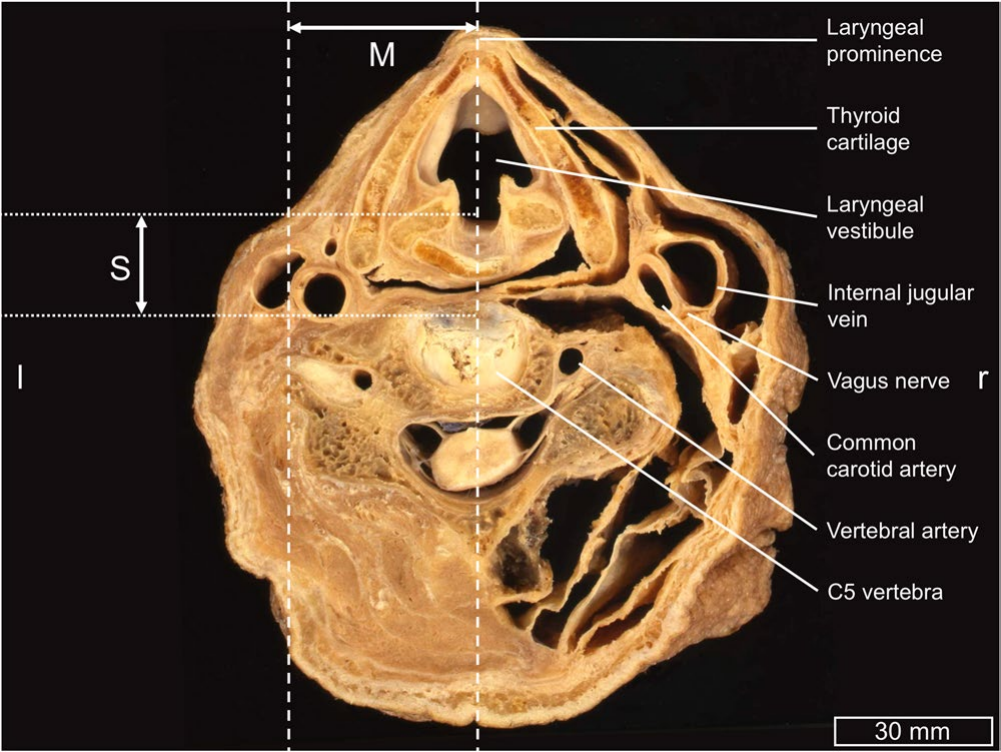
Paraffin-embedded axial-plane section on level of C5 vertebral body. M = midline distance (lateral distance between the laryngeal prominence and the cervical vagus nerve), S = skin distance (anterior-posterior distance between the skin surface and the cervical vagus nerve in sagittal direction), l = left, r = right.

**Figure 2:**
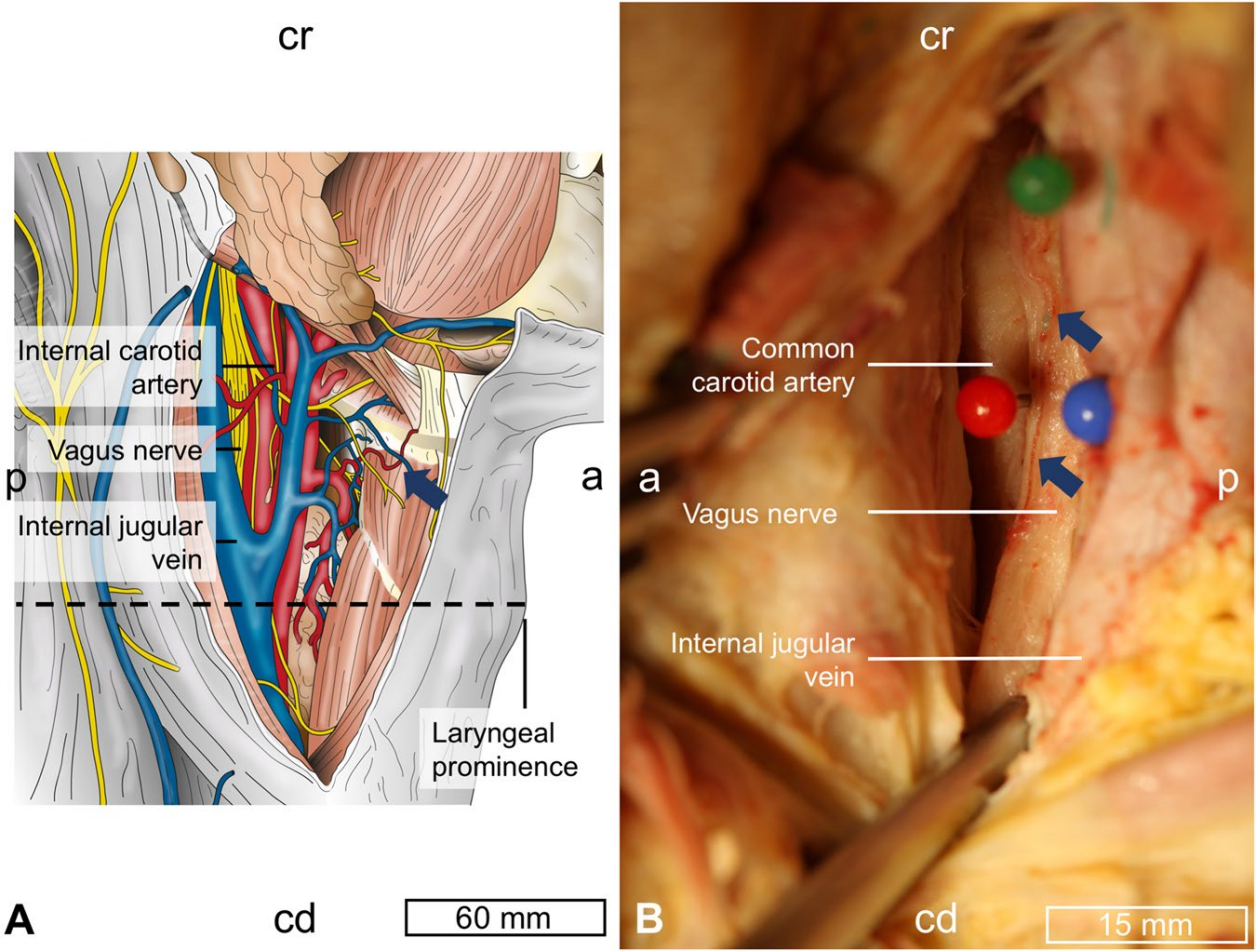
Topography of the neck region. The interrupted line indicates the level at which the cervical vagus nerve was visualized (**A**) on the level of the laryngeal prominence. Vessels external to the epineurium were observed in 25/51 cases (blue arrows, **B**). a = anterior, cd = caudal, cr = cranial, p = posterior.

The CVN is a major site for direct and also an alternative indirect (transcutaneous) stimulation zone to auricular VNS approach, as the nerve is accessible there. In contrast to the increasing number of VNS procedures carried out presently, reliable morphometric data for the CVN in humans is still missing. Older literature suggests that the CVN is a highly consistent structure in the neck region^24–27^, easy to be localized (Figs 1, 2). It has been stated that the CVN is reliably located dorsomedially between the common or internal carotid artery and the internal jugular vein with no branching in this region^24,25,27^. Based on anecdotal descriptions contradicting this consistency we recently investigated both topographical relationships of the CVN and its branching pattern^28,29^. Clear evidence was found that the CVN is highly variable regarding its position in the carotid sheath, with the dorsomedial position existent in less than 50%^29^ and that branching of the CVN in the carotid sheath (nerve branches of the CVN) occurred in one third of all cases^28^, which had not been described in such high rates before.

Recent developments in invasive cervical VNS device design trend towards electrodes, which can be directly clamped on or wrapped around the CVN, and non-invasive devices using a transdermal approach. It has become evident here that further detailed morphological and topographical knowledge of CVN is needed for an optimal surgical approach, the implementation of the VNS devices and/or electrodes. This can potentially contribute to the improvement of the clinical results.

Consequently, the given study aimed at providing extensive morphometric and surface topography data on the CVN in the carotid sheath as reliable baseline data to improve the safety of implanting stimulator electrodes at appropriate sizes, and to cater towards VNS electrode and stimulation mode development. A secondary objective was to determine vascularity of the CVN at various levels superficial to the nerve and in the epineurium, as vascularity not only may influence the stimulation parameters, but potentially be at risk by the surgical procedure or cuff electrodes in form of minor to major bleedings. The following questions were addressed:

1. Which dimensions does the CVN have, and what is the spatial surface topography at the level of the laryngeal eminence?
2. Are the number of CVN fascicles (collection of nerve fibers surrounded by perineurium) or CVN vascularity influenced by age, sex, side or branching patterns?

## Results

Morphometric data including the greater and lesser diameters, mean area as well as skin and midline distance were obtained in all specimens. Branching was observed in 7 of the 51 CVNs (13.7%).

**Figure 3:**
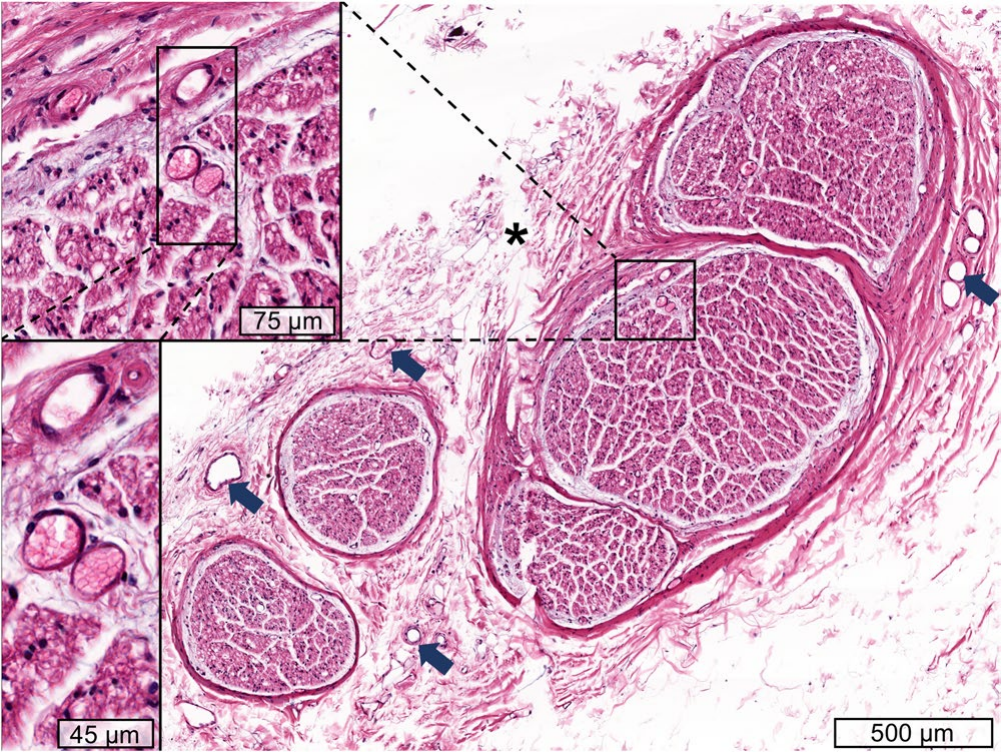
Hematoxylin-eosin stained histology sample of the cervical vagus nerve. A number of fascicles can be seen, separated by perineurium. Subepineural vascular supply could be observed (arrows), as well as subperineural supply (magnifications on left side). *Artefact introduced by the processing of the sample.

The embalming procedure appeared to minimally influence the dimensions and shape of the CVN, confirmed by Cronbach’s α values greater than 0.98 for all parameters, indicating excellent measurement repeatability comparing the fresh vs. fixed CVN dimensions. Equally, comparison of the mean values fresh vs. fixed yielded highly similar values (Supplement Fig. 1). Histology confirmed the existence of nerve tissue (Fig. 3). In line with our previous experiments^28,29^, identification of the branches of the CVN such as the recurrent or non-recurrent branches and the inferior vagus nerve have been identified^30–32^. Detailed data are given in Supplement Tables 1 and 2.

### Skin distance of the CVN appears not to be side-, gender- or branching dependent

Distances were 36.2 ± 9.4 mm from the skin in the anterior-posterior direction, and 34.5 ± 6.2 mm (mean ± standard deviation) from the midline, indicated by the laryngeal eminence. Comparison of the mean morphometry values has shown no differences comparing female to male CVN as well as left to right and branching in the given sample (all *p* > 0.05).

### Nerve dimensions appear to be closely related to skin and midline distances

**Figure 4:**
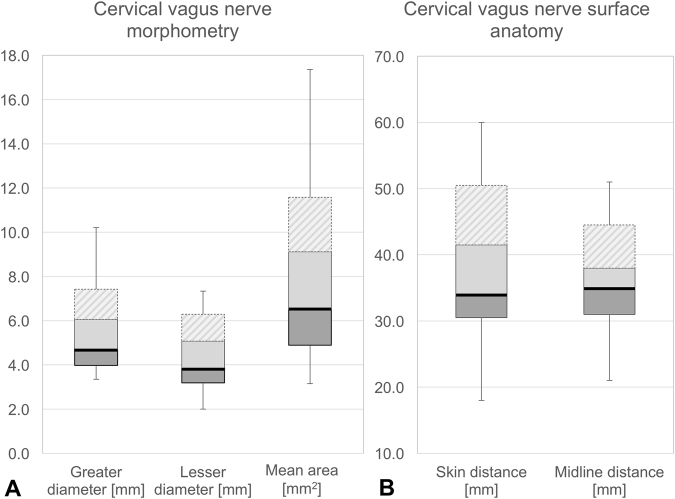
Box plots of cervical vagus nerve morphometry and surface anatomy. Darker grey boxes indicate the 25% percentile, the solid black line the median, the lighter grey box the 75% percentile, and the hatched are the 95% percentile. Whiskers show the minima and maxima. (**A**) Nerve morphometry, (**B**) Nerve relations to the skin surface (skin distance) and laryngeal prominence (midline distance).

The mean values of the CVN over all samples were as follows: 5.1 ± 1.5 mm greater diameter, 4.1 ± 1.3 mm lesser diameter, 7.2 ± 3.1 mm^2^ cross-sectional area (CSA, Supplement Table 1). The greater diameter of the CVN was ≤7.0 mm (7.5 mm), the lesser diameter ≤5.8 mm (6.4 mm), and the area ≤11.0 mm^2^ (11.6 mm^2^) in 90% (95%) of the CVN, respectively (Fig. 4A). Vice versa, only 5% of the CVN have greater diameters between 7.5 and 10.2 mm, lesser diameters between 6.4 and 7.3 mm, and CSAs between 11.0 and 17.4 mm^2^. Skin distance was ≤49.0 mm (51.1 mm) and midline distance ≤41.0 mm (45.5 mm) in 90% (95%) of the CVN, respectively (Fig. 4B). Vice versa, only 5% of the CVN were located deeper than 51.1 to 60 mm and more laterally of the midline than 41.0 to 51.0 mm from the midline.

Age appeared neither to influence nerve morphometry, nor the skin, nor the midline distance of the CVN, except for mean CSA which showed a strong inversely proportional relationship with age (coefficient of correlation = r = −0.28, p = 0.041). The lesser diameter correlated strongly positively with greater diameter (r = + 0.81, p < 0.001). Further positive correlations were found for midline distance and skin distance (r = +0.30, p = 0.030), as well as midline distance and mean CSA (r = +0.38, p = 0.006), indicating that the midline distance is closely related to both the skin distance and the size of the nerve (i.e. “the nerve gets thicker the deeper it is”). Branching of the CVN in the carotid sheath^28^ did not affect the CSA in this given sample.

### CVN fascicle number and vascularity appear not to be influenced by sex, body side or branching

**Figure 5:**
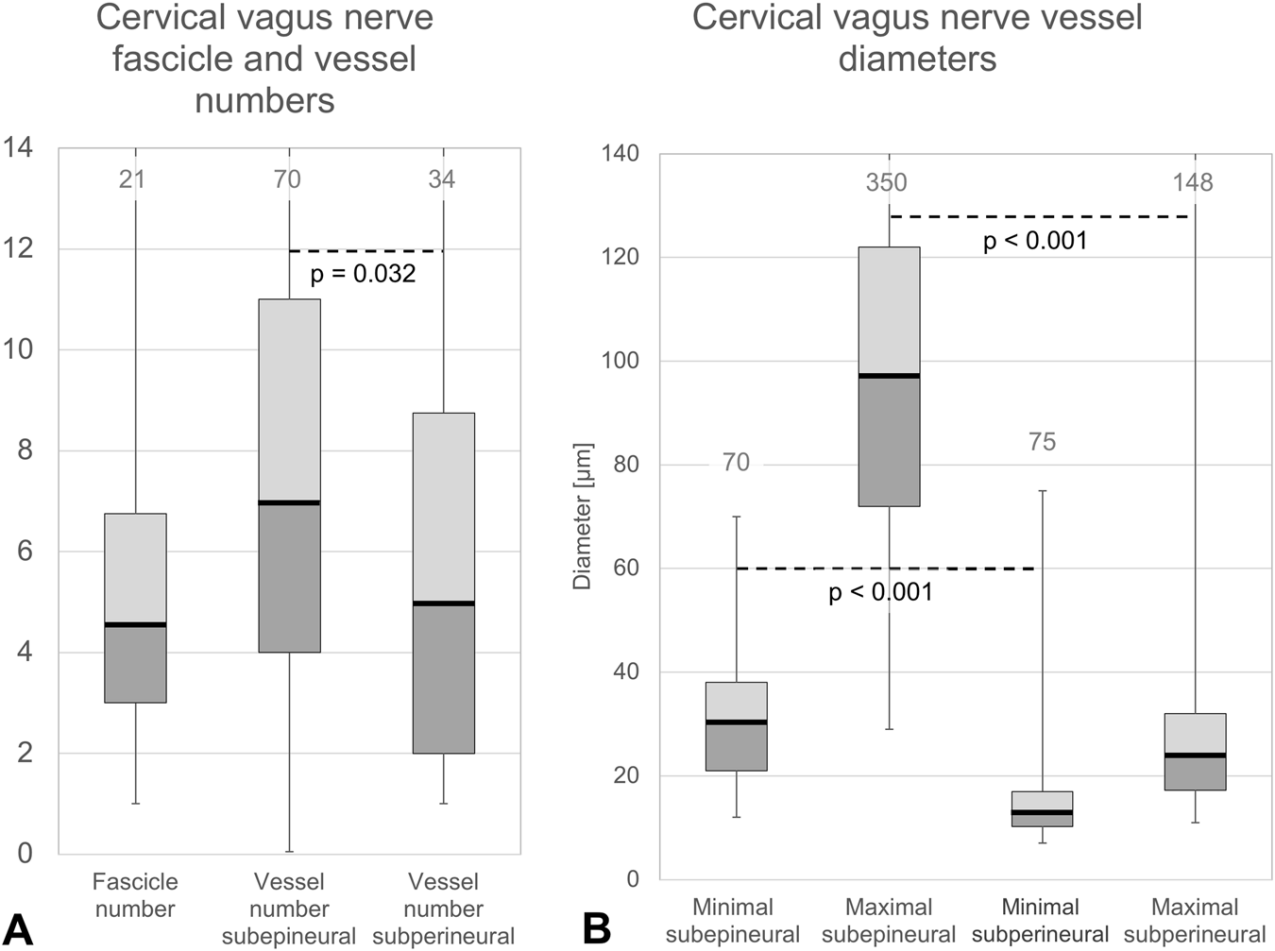
Cervical vagus nerve fascicle and vessel number (**A**) and vessel diameters (**B**). Darker grey boxes indicate the 25% percentile, the solid black line the median, the lighter grey box the 75% percentile, the whiskers minima and maxima. Significant differences were observed comparing the number as well as the minimum and maximum diameters between subepineural and subperineural vessels. The grey numbers on top give the numerical maximum.

**Figure 6:**
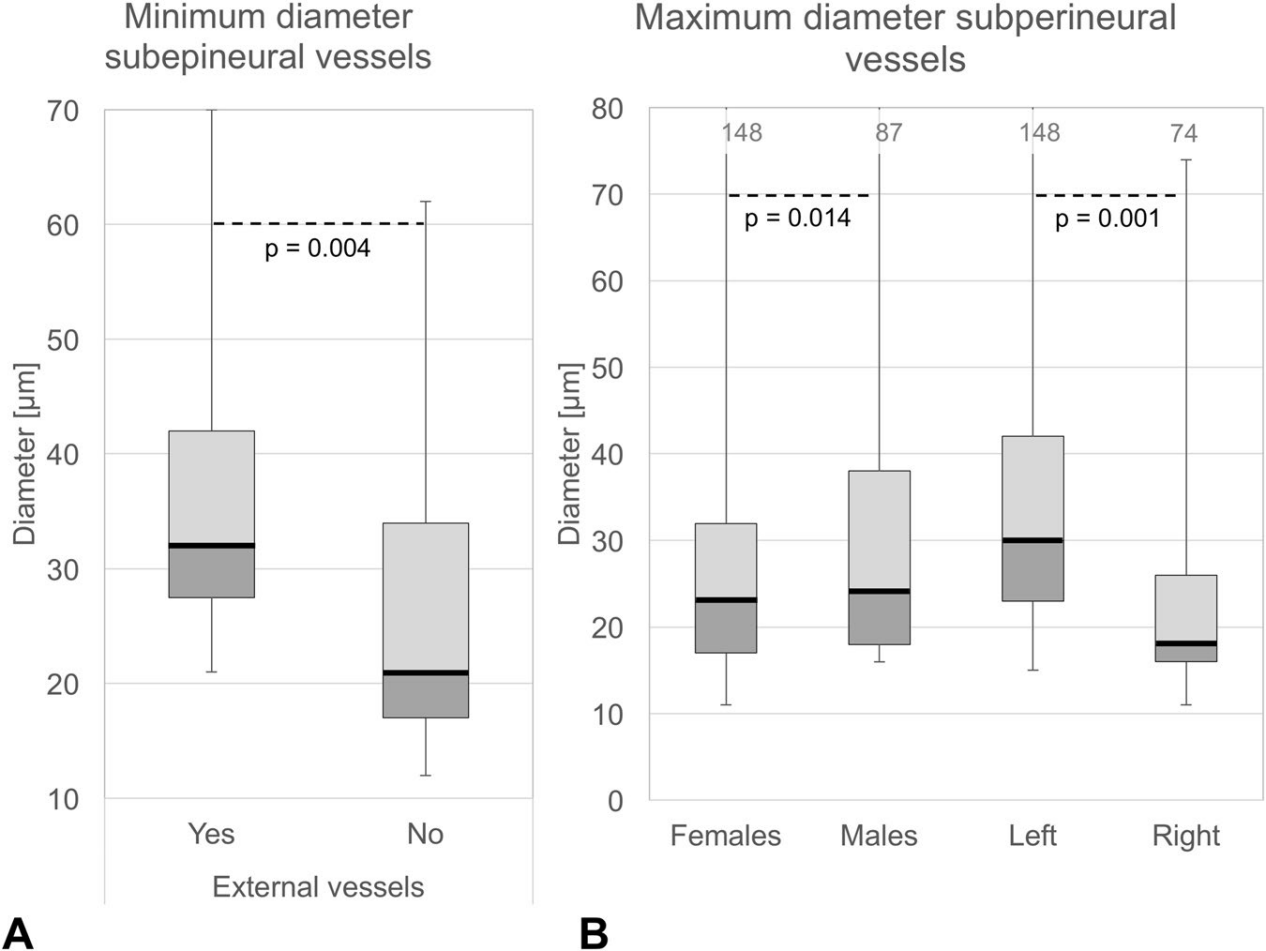
(**A**) Comparison of vessel diameters between cervical vagus nerves with external vessels (relative to the epineurium) showing significant differences. Darker grey boxes indicate the 25% percentile, the solid black line the median, the lighter grey box the 75% percentile, the whiskers minima and maxima. (**B**) Comparison of the maximum diameter of subperineural vessels between sexes and sides, showing both significant differences. Darker grey boxes indicate the 25% percentile, the solid black line the median, the lighter grey box the 75% percentile, the whiskers minima and maxima. The grey numbers on top give the numerical maximum.

A vessel external to the epineurium, the so-called vagal artery, was observed in 25 of the 51 cases (49%, Supplement Table 2). The vagal artery gave off branches continuous with the epineurium, reaching down beyond the exposed site of the incision. The number of CVN fascicles averaged 5.2 ± 3.5, with no differences between males and females, left or right, nor between the CVN with and without branches, as shown in Fig. 5A. Larger values were observed for the numbers of subepineural vessels (within epineurium but outside the perineurium) compared to the subperineural vessels (within the perineurium), regarding number (8.7 ± 6.4 vs. 6.9 ± 7.3; p = 0.032), minimum diameter (30.5 ± 12.9 vs. 16.2 ± 11.8 μm, p < 0.001) and maximum diameter (105.2 ± 55.2 vs. 31.5 ± 25.0 μm; p < 0.001). Average data are given in Supplement Table 2 and box plots representing the medians and quartiles in Fig. 5. Further comparison showed smaller minimum diameters of subepineural vessels in CVN with no external vessels compared to those with vessels (26.2 ± 12.6 vs. 35.0 ± 11.1 μm, p = 0.004; Fig. 6; Supplement Table 2). Larger maximum diameters of subepineural vessel diameters were observed in females compared to males (p = 0.014) and on the left compared to the right side (p = 0.001).

Nerve fascicle number correlated strongly positively with the number of subepineural vessels (r = +0.67, p < 0.001) and moderately positively with the number (r = +0.31, p = 0.05) and maximum diameter of the subepineural vessels (r = +0.42, p = 0.005). Another significant moderately positive correlation was found between number of subepineural vessels and CSA (r = +0.35, p = 0.017), and between nerve fascicle number and CSA (r = +0.40, p = 0.005).

## Discussion

The given study investigated the detailed morphometry, vascularity and surface topography of the CVN for both invasive and non-invasive VNS. Previous findings that the CVN has previously unknown branching patterns^28^, large variations in topography^29^, and a variety of anastomoses^33–36^ indicate that the CVN is not as easy to be visualized surgically, underlining the rationale to provide detailed morphometry to improve surgery, stimulate the nerve reliably and to rule out potential effects of nerve irritation such as focal demyelinization^37^. Both morphometric values and spatial alignment in the neck relative to the laryngeal eminence showed large inter-individual variation, supplemented by novel information on the vascular supply of the CVN.

The CVN has both afferent and efferent roles providing somatic, sympathetic and parasympathetic information^18,25^, with 60–80% being afferent C fibers from the thoracic and abdominal viscera^38^. Efferent fibers include acetylcholine, nitric oxide, vasoactive intestinal peptide, calcitonin gene-related protein as neurotransmitters, and tyrosine-hydroxylase as an enzyme^23^. The CVN contains not only parasympathetic, but also around 5% sympathetic fibers in humans^18^. As the nerve develops, myelin forms progressively^39–41^, to the effect that axon and fiber diameters increase until a certain age^39^. In an adult, approximately 35,100 myelinated and 4400 unmyelinated fibers^40^ can be found. The anatomy of the CVN, being diverse regarding its fiber qualities and projections, makes it an ideal site for electric stimulation, as a number of modulation procedures can be accomplished via one structure.

Stimulation of the vagus nerve has meanwhile evolved into being an established therapy in epilepsy and depression due to its seizure-reducing and psychiatric effects^3,10^. Further anti-inflammatory properties of the CVN have been postulated^7–12^. Apart from the well-studied central projections of the CVN, three pathways appear to be responsible for this effect, namely a hypothalamic-pituitary-adrenal axis facilitating cortisol release, a cholinergic pathway causing reduced tumor necrosis factor (TNF)-α release, and a splenic sympathetic pathway reducing TNF-β release^7,9^. These mechanisms appear to be promising for IBD^11,12,42,43^. Further evidence has been presented regarding decreases cytokines such as TNFα, interleukin (IL)-1β, and IL-6 in patients with rheumatoid arthritis^44^. VNS is commonly performed on the left side, as stimulation of the right CVN has been shown to cause bradycardia, asystole and other cardiac side effects^14,15,20^. We and others could recently show that even intermittent transcutaneous VNS on the right compared to the left side resulted in cardiac parasympathetic activation^45,46^.

In the light of both the greater and the lesser diameter presented in this paper, it became evident that an electrode diameter of 7.5 mm would suit a vast majority of patients. In two thirds of all cases even a diameter of 4.5 mm would suffice for the larger diameter (Supplement Tables 1 and 2). If a design was chosen which allowed to wrap the electrode around the lesser diameter of the nerve, the opening area could be further reduced to an approximate of 6.5 mm, and in two thirds of all cases the opening area would not have to be larger than approximately 4.5 mm. The fact that no sex-, side- or branching related differences appear to exist in the given cohort is indicative that electrode development might not necessarily have to be adapted to these parameters, nor to age, as is the case for the recurrent laryngeal nerve^47^. The results presented in the given were different from our previous study in which we have shown branching-dependent differences in CVN size^28^ and side dependencies observed with high-resolution ultrasound^48^, indicating that further study might be necessary to further refine electrode dimensions. However, moderate age-dependent decreases in the CSA of the CVN were found, in line with the clinical ultrasound data^48^. In the anatomical studies, side and age differences might have been missed due to the elderly study population where axonal degeneration might be quite advanced. Although most of the histological investigations on peripheral nerves demonstrated a progressive reduction of the myelinated fibers with age, at the same time the peri- and epineural sheaths were reported to thicken with age^49^, which could partially explain our results being larger than previous reports on CVN dimensions. In this context, the diameter of the VNS could potentially remain in the same range but the intensity of the stimulation might need to be adjusted by age independent of potential scarring. An important factor of bias mentioning here is the age of our specimen population, which is significantly higher than patients who would potentially undergo VNS procedures.

Both skin distance and midline distance, indicated by the anterior-posterior depth under the dermis, and the distance from the laryngeal eminence appear to be in the range of approximately 35 mm, which may serve as a gross reference value when localizing the CVN for a targeted depth in case of a transcutaneous stimulation approach, as well as the open surgical one. Though, again, large variations exist for the surface topography of the CVN, it appears that it is highly unlikely to find the nerve deeper than 50 mm from the surface and the laryngeal eminence. These data may have implications for both surgical and transcutaneous VNS.

Another important consideration for refining electrodes at the CVN for VNS is its vascularity. The number of vagal arteries in the sample of this study is much smaller than reported previously^47^; 49%, were confirmed here by histology. The advanced age population of the present study may also underline the smaller number of vagal arteries, though in our sample no correlations were found for age and vascularity. Progressive scarring of the vasa nervorum and impaired blood flow to nerves by aging has also been shown^50^. It has been demonstrated that the vessels supplying the extracranial part of the CVN originate of the inferior thyroid artery^47,51^, the internal carotid, posterior meningeal artery, as well as the vertebral and esophageal artery^51,52^. Though the exposure in our specimens has been kept to the size of the surgical intervention, it could be shown that the numbers of subepineural and subperineural vessels in the CVN was larger than the number of nerve fascicles, forming a rich vascular network supplying the nerve at the different levels relative to the neural coverings. The vascularity of the CVN is not only of interest for stimulation-related (side) effects, potentially caused by stimulation effects on the smooth muscle cells, but also for the direct branches of the CVN receiving their supply from the arterial stem of the vagal artery. The arterial supply might become affected as a consequence of the surgical approach to the CVN. Laryngopharyngeal symptoms including hoarseness, dyspnea and dysphagia^10,53–55^ could also be the consequence of interrupted blood supply to the recurrent laryngeal nerve supplied by the vagal artery^47^. Based on the present data together with previous studies^28,29^ surgical approach to the CVN must be performed with caution and precise knowledge of CVN morphometry and topography. For the vasculature of the CVN, moreover, differences were observed between the size of subepineural vessels in those nerves with and without the presence of a vagal artery, as well as differences in vessel dimensions, sex and side. Such differences may have relevance for potential compensatory mechanisms of the CVN following vagal artery transection, as well as implications for the maximum blood supply of the CVN. However, due to lacking functional data on blood flow, further conclusions on this would be largely hypothetical. Of further interest, increasing fascicle numbers was related to a higher number of subepineural vessels, both adding to increased CSA of the nerve.

A number of misconceptions appear to still exist for the CVN regarding its “constant” anatomy, namely that (1) the CVN has no branches in the lower cervical area, (2) is Iocalized posteromedially in the carotid sheath between the (internal) carotid artery and the internal jugular vein, and that (3) the CVN is a parasympathetic-only nerve. While none of these statements is incorrect for a majority of CVNs, marked variation exists, justifying a detailed description of morphometric and topographic anatomy. It appears that the CVN can be visualized in a 35 × 35 mm distance lateral of the laryngeal eminence and posterior to the skin of the neck. Moreover, this study gave a detailed morphometric description of CVN dimensions and vascularity, as a basis for further developments in VNS.

A couple of limitations need to be addressed for the given study. First, a larger and more heterogeneous sample size regarding age distribution would have been preferential to study potential differences in more detail and to be closer to the study population receiving VNS. Shrinkage and deformation related to the embalming procedure of the cadaver and the embedding procedure of the tissues are further potential shortcomings, though for the step of ethanol-glycerin embalming used here the changes induced in nerve dimensions and shape were minute. Furthermore, due to the anatomical (basic science) nature of this study, it was impossible to provide clinical proof from our findings on basis of the same tissues. Future studies might need to address these limitations in larger sample sizes, potentially quantifying fiber counts and qualities in more detail in the context of branching and the side-dependent innervation fields of the CVN.

## Materials and Methods

All body donors while alive gave written and informed consent to the donation of their corpses after their death for teaching and research purposes. Institutional approval for the use of the post-mortem tissues was obtained from the Institute of Anatomy, University of Leipzig, in line with the Saxonian Death and Funeral Act of 1994 (third section, paragraph 18 item 8) as part of an ongoing donation program. All experiments have been conducted according to the principles of the Declaration of Helsinki.

### Preparation and measurements

The CVN was identified in the region of the carotid triangle in 27 human necks (16 females, 11 males), on basis of a similar sample as in Hammer *et al*. and Planitzer *et al*.^28,29^. 51 CVN were examined in total, 24 bilaterally, two on the left and one right side (contralateral side each allocated to teaching). The mean age at death was 88.1 ± 6.6 years (range 73 to 103 years). Anatomical fixation and conservation was carried out using an ethanol-glycerin protocol^56,57^. The exposure to the CVN was performed in an adapted manner as done when implanting VNS electrodes for treating epilepsy^58^. The skin and the underlying platysma were incised, and this incision was further extended to the medial border of the sternal head of the sternocleidomastoid muscle. Superficially, the extent of the incision reached from the level of the laryngeal prominence to the superior belly of the omohyoid muscle, and then was extended to the posterior belly of the digastric muscle. Structures at risk were identified and preserved with a particular focus on the ansa cervicalis^59^, the (non-) recurrent recurred laryngeal nerve^60–62^ and larger vessels; the anterior jugular vein was partly removed to enhance the exposure. The carotid sheath was opened ventrally until the carotid artery was visualized, followed by the internal jugular vein, and the CVN at the given length with its branches. Care was taken during the dissection not to distort the spatial relationships. Distances from the midline, on the level of the laryngeal eminence (midline distance) and the anterior-posterior distance from the skin surface in sagittal direction (skin distance) were obtained using a digital vernier caliper (PMS150, Conrad Electronic SE, Hirschau, Germany; accuracy 0.01 ± 0.005 mm), as shown in Figs 1 and 2A, and an image taken from the incised neck with the CVN, as shown in Fig. 2B. Tissue samples were obtained following casting *in situ* to determine the nerves’ CSAs and for histological validation of nerve tissue using hematoxylin-eosin. Further details on the casting procedure can be found in Hammer *et al*.^63^ Histology slides were scanned with an Aperio CS2 microscope under 400x magnification, as seen in Fig. 3. Arteries were identified morphologically on basis of their wall features, and their diameters computed using the Aperio ImageScope Software (both Leica Biosystems, Wetzlar, Germany), focusing on vessels with ≥7 μm as a threshold between arteriole and capillary^64^. Three layers of vessels were investigated:

1. *subperineural*
2. *subepineural*
3. *external* (outside of the epineurium and adjacent to the CVN), i.e. vagal artery.

Further four CVN were harvested in a fresh and anatomically unfixed condition, casted, fixed with ethanol glycerin according to the above-mentioned protocol, and re-measured to determine the alterations in size and shape induced by the fixation.

### Statistical analysis

Microsoft Excel 2016 (Redmond, WA, USA) and SPSS 23.0 (IBM, IL, USA) were used for statistical evaluation. The Kolmogorov-Smirnov test was applied to determine normal distribution, followed by the Mann-Whitney U-test, Wilcoxon signed-ranks or Kruskal-Wallis test as non-parametric tests to determine gender-, side- and branching-related differences in the measurements of the CVN cross section and distance skin surface and midline distance as well as differences induced by fixation, respectively. Cronbach’s α was used to assess the test-retest reliability for the casts and reliability of the measurements following the casting of the CVN. The Spearman test was utilized to determine correlations. *P values* of 0.05 or less were considered as statistically significant.

## Electronic supplementary material

Supplementary Information
