# Supplementary material for: Cervical vagus nerve morphometry and vascularity in the context of nerve stimulation - A cadaveric study
Source: Sci Rep. 2018 May 22;8:7997. doi: 10.1038/s41598-018-26135-8 (PMC5964190; doi:10.1038/s41598-018-26135-8)
Supplement: Supplementary file 1 — Supplementary Information [file 41598_2018_26135_MOESM1_ESM.pdf]

Cervical vagus nerve morphometry and vascularity in the context of nerve stimulation - A cadaveric study

Niels Hammer [1*], Sabine Löffler [2], Yusuf Cakmak [1], Benjamin Ondruschka [3], Uwe Planitzer [4], Michael Schultz [5,6], Dirk Winkler [4], David Weise [7]

[1] Department of Anatomy, University of Otago, Dunedin, New Zealand

[2] Department of Anatomy, University of Leipzig, Germany

[3] Institute of Legal Medicine, University of Leipzig, Germany

[4] Department of Neurosurgery, University of Leipzig, Germany

[5] Department of Medicine, Dunedin School of Medicine, University of Otago, Dunedin, New Zealand

[6] Gastroenterology Unit, Southern District Health Board, Dunedin Hospital, Dunedin, New Zealand

[7] Department of Neurology, University of Leipzig, Germany

Corresponding author

Niels Hammer, M.D., Dr. habil., Department of Anatomy, University of Otago, Lindo Ferguson Building, 270 Great King St, Dunedin 9016, New Zealand; Phone: +64 3 479 7362, Fax: +64 3 479 7254

Email: nlshammer@googlemail.com

Key words

anatomical landmark, carotid sheath, cervical vagus nerve, electrical stimulation, epilepsy, nerve morphometry, surface topography, vagus nerve stimulation

Supplement figure 1

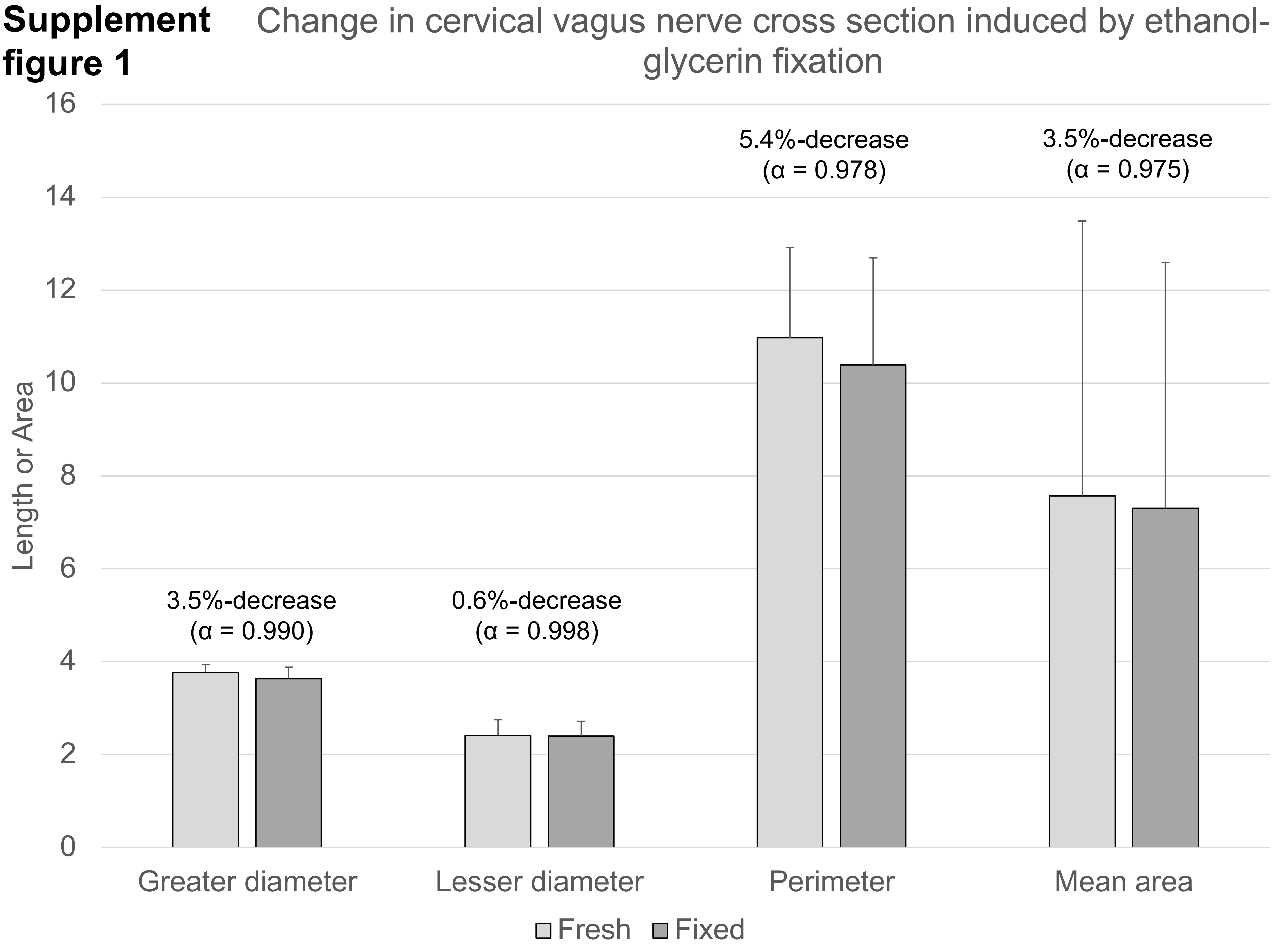

Supplement table 1

n=51	Age [years]	Sex	Side	Branching	Greater diameter [mm]	Lesser diameter [mm]	Cross-sectional area [mm^2]	Skin distance [mm]	Midline distance [mm]
**All**	88.1 ± 6.6				5.1 ± 1.5	4.1 ± 1.3	7.2 ± 3.1	36.2 ± 9.5	34.5 ± 6.2
**Maximum**	103				10.2	7.3	17.4	60.0	51.0
**Minimum**	73				3.4	2.0	3.2	18.0	21.0
**Females**	88.4 ± 6.9	16			4.8 ± 1.4	3.9 ± 1.1	6.6 ± 2.6	36.5 ± 10.1	34.1 ± 6.4
**Males**	87.6 ± 5.8	11			5.6 ± 1.6	4.4 ± 1.6	8.1 ± 3.3	35.9 ± 8.2	35.0 ± 5.7
**Left**	88.0 ± 6.6		26		5.2 ± 1.7	4.0 ± 1.4	7.3 ± 3.6	36.8 ± 8.5	34.2 ± 6.1
**Right**	88.1 ± 6.3		25		5.0 ± 1.3	4.2 ± 1.1	7.2 ± 2.4	35.6 ± 10.1	34.8 ± 6.2
**No branching**	89.0 ± 5.9			44	5.3 ± 1.6	4.2 ± 1.3	7.5 ± 3.1	36.1 ± 9.3	35.0 ± 6.0
**Branching**	86.5 ± 7.2			7	4.1 ± 0.4	3.4 ± 0.8	5.4 ± 1.3	36.7 ± 9.5	31.6 ± 6.4
**95-percentile**					7.5	6.4	11.6	51.1	45.5
**90-percentile**					7.0	5.8	11.0	49.0	41.0
**67-percentile**					4.5	4.3	7.0	38.8	36.0
**50-percentile**					4.8	3.8	6.5	36.0	35.0

Supplement table 2

n=51	Age [years]	Number Fascicles	External vessel	Subepineural vessels			Subperineural vessels		
				Number	Minimum diameter [µm]	Maximum diameter [µm]	Number	Minimum diameter [µm]	Maximum diameter [µm]
**All**	88.1 ± 6.6	5.2 ± 3.5		8.7 ± 6.4[†]	30.5 ± 12.9[¶]	105.2 ± 55.2[¢]	6.9 ± 7.3[†]	16.2 ± 11.8[¶]	31.5 ± 25.0[¢]
**Maximum**	103	21.0		31.0	70.0	350.0	34.0	75.0	148.0
**Minimum**	73	1.0		0.1	12.0	29.0	1.0	7.0	11.0
**Females**	88.4 ± 6.9	4.2 ± 2.1		7.5 ± 4.8	32.2 ± 14.0	92.8 ± 37.5	6.9 ± 7.5	17.2 ± 13.7	31.1 ± 27.6[*]
**Males**	87.6 ± 5.8	6.6 ± 4.4		10.4 ± 7.8	27.7 ± 9.7	125.8 ± 70.0	7.0 ± 6.7	14.8 ± 7.2	32.0 ± 19.7[*]
**Left**	88.0 ± 6.6	5.1 ± 4.1		8.5 ± 7.1	32.4 ± 14.8	102.3 ± 37.0	5.6 ± 4.0	18.4 ± 14.2	40.0 ± 29.9[+]
**Right**	88.1 ± 6.3	5.2 ± 2.6		8.8 ± 5.4	28.7 ± 10.0	108.1 ± 67.1	8.3 ± 9.2	14.0 ± 7.7	22.9 ± 13.4[+]
**No branching**	89.0 ± 5.9	5.5 ± 3.6		8.9 ± 6.7	31.6 ± 13.0	110.0 ± 56.8	7.0 ± 7.6	16.3 ± 12.2	32.6 ± 26.4
**Branching**	86.5 ± 7.2	3.2 ± 1.1		6.8 ± 2.1	23.5 ± 7.2	74.5 ± 17.7	6.5 ± 4.6	15.8 ± 7.5	24.8 ± 5.5
**External vessel yes**	88.2 ± 6.7	4.7 ± 2.6	25	7.5 ± 5.1	35.0 ± 11.1[#]	110.1 ± 63.1	7.9 ± 7.6	18.2 ± 15.4	38.5 ± 31.2
**External vessel no**	88.1 ± 5.9	5.6 ± 4.0	26	9.8 ± 7.1	26.2 ± 12.6[#]	100.6 ± 44.6	6.1 ± 6.8	14.6 ± 6.6	25.7 ± 15.3
